# Relationship between caffeine intake and thyroid function: results from NHANES 2007–2012

**DOI:** 10.1186/s12937-023-00866-5

**Published:** 2023-07-26

**Authors:** Jiaping Zheng, Xinyan Zhu, Guiqing Xu, Xingchen Wang, Mengyang Cao, Shusen Zhu, Rui Huang, Yu Zhou

**Affiliations:** 1 Department of Rehabilitation Medicine, School of Health, Fujian Medical University, Fuzhou, China; 2 Department of Clinical Pharmacy and Pharmacy Administration, School of Pharmacy, Fujian Medical University, Fuzhou, China; 3 Department of Intelligent Medical Engineering, School of Medical Imaging, Fujian Medical University, Fuzhou, China; 4 Fujian Normal University Hospital, Fuzhou, China

**Keywords:** Caffeine, Thyroid, Metabolism, NHANES

## Abstract

**Background:**

Moderate caffeine intake decreases the risk of metabolic disorders and all-cause mortality, and the mechanism may be related to its ergogenic actions. Thyroid hormones are vital in metabolic homeostasis; however, their association with caffeine intake has rarely been explored.

**Objective:**

To investigate the association between caffeine intake and thyroid function.

**Methods:**

We collected data on demographic background, medical conditions, dietary intake, and thyroid function from the National Health and Nutrition Examination Survey (NHANES) 2007–2012. Subgroups were classified using two-step cluster analysis, with sex, age, body mass index (BMI), hyperglycemia, hypertension, and cardio-cerebral vascular disease (CVD) being used for clustering. Restrictive cubic spline analysis was employed to investigate potential nonlinear correlations, and multivariable linear regression was used to evaluate the association between caffeine consumption and thyroid function.

**Results:**

A total of 2,582 participants were included, and three subgroups with different metabolic features were clustered. In the most metabolically unhealthy group, with the oldest age, highest BMI, and more cases of hypertension, hyperglycemia, and CVD, there was a nonlinear relationship between caffeine intake and serum thyroid stimulating hormone (TSH) level. After adjusting for age, sex, race, drinking, smoking, medical conditions, and micronutrient and macronutrient intake, caffeine intake of less than 9.97 mg/d was positively associated with serum TSH (*p* = 0.035, standardized *β* = 0.155); however, moderate caffeine consumption (9.97–264.97 mg/d) indicated a negative association (*p* = 0.001, standardized *β* = − 0.152).

**Conclusions:**

Caffeine consumption had a nonlinear relationship with serum TSH in people with metabolic disorders, and moderate caffeine intake (9.97 ~ 264.97 mg/d) was positively associated with serum TSH.

**Supplementary Information:**

The online version contains supplementary material available at 10.1186/s12937-023-00866-5.

## Introduction

Caffeine (1, 3, 7-trimethylxanthine) is present in various beverages and foods consumed worldwide. However, the effects of caffeine on human health have not been adequately explored. Clinical and epidemiological evidence supports a positive association between caffeine consumption and reduced all-cause mortality as well as a decreased risk of chronic diseases, including diabetes mellitus, cardiovascular disease, and chronic liver diseases [1–3]. In particular, caffeine has ergogenic effects on systemic metabolism [4–6] and alters glucose and lipid metabolism [7, 8]. Moderate caffeine intake (< 400 mg/day) is recommended in adults for physical and mental health benefits, although the mechanism has not been fully clarified [9].

Caffeine absorption, distribution, and metabolism are associated with various metabolic and lifestyle factors. After absorption, caffeine is mainly metabolized in the liver, with several cytochrome P-450(CYP) isoforms (CYP1A2, CYP2E1, CYP2D6-Met, and CYP1A1) being responsible for its primary alterations, generating several biologically active metabolites (i.e., paraxanthine, theobromine, and theophylline) [10]. Notably, the genetic background partly accounts for the inter-individual variation in caffeine metabolism, as well as metabolic disorders, drinking, and smoking habits. Obesity mildly modifies caffeine pharmacokinetics [11, 12], and altered patterns of caffeine and its main downstream metabolites have been observed in diabetic patients with hypoglycemia [13]. Furthermore, alcohol and smoking have been shown to affect caffeine clearance by regulating CYP activity [14, 15].

Thyroid hormones are important regulators of systemic metabolism and neurological development. Thyroid dysfunction and metabolic disorders are closely linked [16]. The National Health and Nutrition Examination Survey (NHANES) III study revealed that the prevalence of thyroid dysfunction was higher in individuals with diabetes [17]. Furthermore, the progression of thyroid disease is exacerbated by metabolic disorders and vice versa [18].

Nutrition is another major factor influencing thyroid function. Numerous studies have reported on effects of certain minerals and vitamins on the thyroid. Iodine deficiency is related to hypothyroidism and goiter, whereas excess iodine may also cause thyroid dysfunction [19, 20]. Selenium supplementation reduces autoimmune responses and alleviate Hashimoto’s thyroiditis [21]. Other micronutrients, including Vitamin D, zinc, and iron, also influence thyroid function and have been discussed extensively elsewhere [22, 23]. However, few studies have analyzed the relationship between caffeine intake and thyroid function, both of which exert metabolic regulatory effects and are closely related to metabolic disorders [24].

In this study, we analyzed data from the NHANES 2007–2012 and stratified the participants with different metabolic features into subgroups. This study aimed to highlight the complex interaction between caffeine intake and thyroid function. We hypothesized that caffeine might be potentially protective against thyroid dysfunction.

## Study population and design

**Fig. 1:**
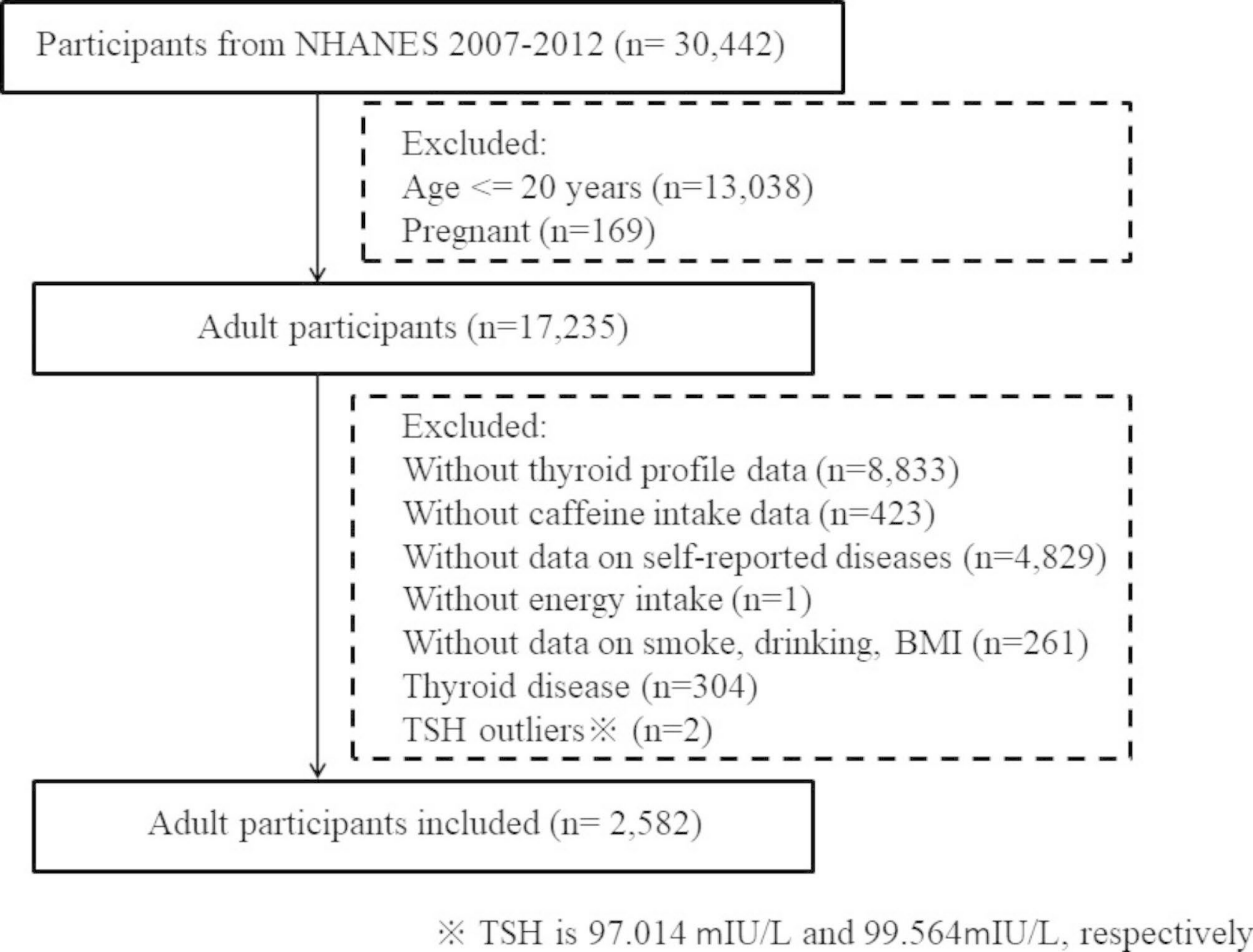
Inclusion and Exclusion Criteria of the Study

NHANES is directed by the United States National Center for Health Statistics to assess the health and nutritional status of adults and children in the United States. Health interviews are conducted in respondents’ homes, and health measurements are performed in specially designed and equipped mobile centers. An advanced computer system using high-end servers, desktop PCs, and wide-area networking collects and processes all of the NHANES data. This observational study used data from three NHANES surveys: 2007–2008, 2009–2010, and 2011–2012. The inclusion and exclusion criteria are shown in Fig. 1. In total, there were 30,442 participants in the NHANES 2007–2012. Participants under 20 years of age or who were pregnant were excluded from the study because the survey only focused on participants aged 20 years and above and their thyroid issues. Furthermore, participants were excluded if they lacked thyroid profile data, caffeine intake data, or self-reported disease information. Missing data for cluster analysis, such as BMI, cardiovascular disease, hypertension, drinking, and smoking habits, also led to the exclusion of participants (n = 261). Additionally, one participant was excluded from the diet survey due to reporting no energy intake. A total of 306 participants were excluded due to self-reported thyroid disease (n = 304) or extremely high TSH levels (n = 2) beyond the 99% population distribution. The TSH levels for these two participants were 97.014 and 99.564 mIU/L, respectively.

For the remaining 2,582 cases, demographic data, medical conditions, smoking and drinking habits, diet intake data, and serum thyroid profiles were collected. Race/ethnicity was categorized as Mexican American, non-Hispanic White, non-Hispanic Black, and others (including other Hispanic and other races). Medical conditions were evaluated by asking participants questions such as “Has a doctor or other health professional ever told you that you had a certain disease?” Diabetes was defined as any self-reported diagnosis of diabetes or self-reported use of insulin or anti-diabetic drug. Participants with diabetes or borderline diabetes were further categorized as hyperglycemic. Participants were categorized as having cardio-cerebral vascular disease (CVD) if they had any of the following self-reported conditions: congestive heart failure, coronary heart disease, angina pectoris, heart attack, or stroke.

### Dietary data

Dietary data were derived from the initial 24-hour dietary intake interview conducted on the first day. This interview was conducted in person at the Mobile Examination Center and utilized the Automated Multiple Pass Method (AMPM), the dietary data collection instrument developed by the USDA (http://www.ars.usda.gov/ba/bhnrc/fsrg) [25–27]. USDA’s Food and Nutrient Database for Dietary Studies was used for processing and calculating nutrient intakes, including macro-nutrients and micro-nutrients, and the details of the database are described at http://www.ars.usda.gov/ba/bhnrc/fsrg. We calculated the total intake of macronutrients (carbohydrates, fat, and protein) and micronutrients that might affect thyroid function, including caffeine, selenium, calcium, zinc, magnesium, iron, and vitamin D. There was an enormous amount of missing data on iodine consumption; however, most dietary iodine is excreted in the urine within 24 h of ingestion; therefore, urinary iodine could be considered as a reference for an individual’s daily iodine intake [19]. Therefore, we used the urinary iodine level as a covariate.

### Thyroid profile and biochemical tests

Thyroid profile data were collected, including TSH, free T3 (FT3), free T4 (FT4), thyroglobulin antibody (TgAb), and TPOAb. They were measured in the serum using immune-enzymatic assays, described in detail elsewhere (http://wwwn.cdc.gov/nchs/nhanes/2007-2008/THYROD_E.htm). Serum samples were also analyzed for total high-density lipoprotein (HDL) cholesterol, total low-density lipoprotein (LDL) cholesterol, total cholesterol, and triglycerides.

### Statistical analysis

Micronutrient factors (caffeine, calcium, selenium, iodine, iron, vitamin D, magnesium, and zinc) and thyroid profiles (TSH, FT3, and FT4) were log-transformed to improve their normality. When appropriate, continuous variables were presented as means (standard error) or medians (percentile 25-percentile 75). Categorical variables were presented as frequencies (%). ANOVA and post hoc tests between groups (Bonferroni correction) were used to compare normally distributed variables, while the Kruskal–Wallis test and Bonferroni correction were adopted for variables with a skewed distribution. The chi-square test or Fisher’s exact test was used to compare the categorical variables.

Numerous studies have verified the association of thyroid hormones with metabolic disorders and CVD; drinking and smoking habits also impact thyroid function [28, 29]. Furthermore, caffeine’s metabolism, clearance, and pharmacokinetics are affected by age, sex, metabolic factors, smoking, and diet [10]. Therefore, we applied cluster analysis to stratify the participants into subgroups with different metabolic features. We chose age, body mass index (BMI), sex, hypertension, hyperglycemia, CVD, drinking, and smoking for cluster analysis. A two-step clustering method accommodated categorized and continuous variables in which the optimal clustering number was automatically determined [30].

Multivariable linear regression was used to test the correlation between caffeine intake and serum TSH levels. Restricted cubic spline (RCS) analysis was used to evaluate their nonlinear relationship while adjusting for covariates such as sex, age, BMI, drinking and smoking, diabetes, hypertension, cancer, comorbidity, and other food ingredients. For RCS, knots were placed at 5%, 35%, 65%, and 95% of caffeine intake for both the overall and subgroup analyses. Nonlinear curves were drawn, and piecewise linear regression was performed.

For sensitivity analysis, we applied two evaluations. First, multiple imputations with 5 data sets were used to assess the influence of missing data. Second, participants with abnormal TSH (< 0.1 mU/L or > 10 mU/L) were further excluded from analysis, and the associations between caffeine and thyroid profiles were tested.

Stata SE 16.0 and R 4.4.2 (R Project for Statistical Computing) were used for the regression analysis and RCS drawing. IBM SPSS Statistics 25.0 was used for two-step clustering. Statistical significance was set at *p* < 0.05.

## Results

A total of 2,582 participants were initially included in the study. The baseline characteristics of participants are listed in Table 1. The 1st and 2nd quartiles of caffeine intake groups exhibited younger participants, more females, fewer drinkers and smokers, and lower serum lipid levels than did the other quartiles. Thyroid functions did not differ among subgroups. Additionally, there was a correlation between caffeine consumption and participants’ dietary habits, with individuals consuming higher levels of caffeine also exhibiting increased intake of macro-nutrients such as energy, protein, carbohydrates, and fat.

**Table 1:** Participant Baseline Characteristics

	Dietary caffeine intake				
	Q1 ( < = 13 mg/d)	Q2 (13 ~ 98.8 mg/d)	Q3 (98.5 ~ 221 mg/d)	Q4 ( > = 221 mg/d)	*p*
N	649	642	647	644	
Age (years)	46.00 (31.00, 62.00)	45.00 (31.00, 61.00)	49.00 (36.00, 63.00)	50.00 (38.00, 62.00)	< 0.001
Race					
Mexican American	101 (15.6%)	116 (18.1%)	101 (15.6%)	65 (10.1%)	< 0.001
Non-Hispanic White	196 (30.2%)	207 (32.2%)	274 (42.3%)	420 (65.2%)	
Non-Hispanic Black	238 (36.7%)	167 (26%)	111 (17.2%)	58 (9%)	
Others	114 (17.6%)	152 (23.7%)	161 (24.9%)	101 (15.7%)	
Sex (Female)	305 (47%)	305 (47.5%)	291 (45%)	260 (40.4%)	0.041
Education					
<=High School diploma	329 (50.7%)	309 (48.3%)	303 (46.9%)	300 (46.6%)	0.437
>High School diploma	320 (49.3%)	331 (51.7%)	343 (53.1%)	344 (53.4%)	
Marriage					
Married /Living with partner	343 (52.9%)	385 (60%)	408 (63.1%)	412 (64%)	< 0.001
Widowed/Separated /Divorced	155 (23.9%)	116 (18.1%)	127 (19.6%)	149 (23.1%)	
Never married	151 (23.3%)	141 (22%)	112 (17.3%)	83 (12.9%)	
Smoke					
< 100 cigarretes	554 (85.4%)	538 (83.8%)	503 (77.7%)	444 (68.9%)	< 0.001
>= 100 cigarretes	95 (14.6%)	104 (16.2%)	144 (22.3%)	200 (31.1%)	
Drink					
< 12 drinks/year	194 (29.9%)	169 (26.3%)	152 (23.5%)	116 (18%)	< 0.001
>= 12 drinks/year	455 (70.1%)	473 (73.7%)	495 (76.5%)	528 (82%)	
BMI (kg/m^2^)	27.96 (24.20, 32.80)	27.43 (24.17, 32.08)	27.80 (24.33, 32.52)	28.18 (24.80, 32.39)	0.404
Hyperglycemia	86 (13.3%)	89 (13.9%)	109 (16.8%)	83 (12.9%)	0.160
Hypertension	228 (35.1%)	218 (34%)	242 (37.4%)	214 (33.2%)	0.417
CVD	60 (9.2%)	59 (9.2%)	61 (9.4%)	52 (8.1%)	0.827
Cancer	51 (7.9%)	49 (7.7%)	57 (8.8%)	71 (11%)	0.127
Comorbidities					
0	420 (64.7%)	386 (60.1%)	380 (58.7%)	356 (55.3%)	0.030
< 3	214 (33%)	233 (36.3%)	243 (37.6%)	260 (40.4%)	
>=3	15 (2.3%)	23 (3.6%)	24 (3.7%)	28 (4.3%)	
Serum lipids					
LDL (mmol/L)	2.90 (2.30, 3.41)	2.90 (2.35, 3.58)	3.05 (2.40, 3.70)	3.08 (2.46, 3.70)	0.046
HDL (mmol/L)	1.32 (1.09, 1.60)	1.29 (1.06, 1.53)	1.27 (1.06, 1.55)	1.25 (1.06, 1.55)	0.163
Total cholesterol (mmol/L)	4.84 (4.22, 5.61)	4.89 (4.19, 5.59)	5.07 (4.32, 5.79)	5.09 (4.40, 5.83)	< 0.001
Thyroid function					
TSH (mU/L)	1.50 (1.02, 2.23)	1.48 (1.04, 2.12)	1.43 (1.03, 2.07)	1.56 (1.11, 2.28)	0.140
FT3 (pg/mL)	3.12 (2.90, 3.40)	3.18 (2.96, 3.41)	3.15 (2.94, 3.41)	3.17 (2.95, 3.40)	0.243
FT4 (pmol/L)	10.30 (9.00, 11.50)	10.30 (9.30, 11.50)	10.30 (9.30, 11.60)	10.30 (9.00, 11.50)	0.281
TPOAb (U/mL)	0.60 (0.30, 1.3)	0.60 (0.30, 1.60)	0.60 (0.30, 1.40)	0.60 (0.30, 1.40)	0.499
Diet					
Energy (kcal/d)	1855.00 (1328.00, 2455.00)	1932.00 (1402.00, 2553.00)	1989.00 (1512.50, 2643.50)	2158.50 (1650.60, 2910.00)	< 0.001
Protein (gm/d)	72.66 (50.57, 99.05)	73.44 (50.16, 96.94)	77.86 (53.52, 102.75)	81.08 (57.23, 113.02)	< 0.001
Carbohydrate (gm/d)	221.83 (164.04, 294.95)	239.40 (177.41, 318.37)	240.70 (186.15, 332.27)	260.88 (191.57, 345.20)	< 0.001
Fat (gm/d)	64.28 (40.41, 96.49)	64.71 (45.22, 99.0)	73.18 (49.68, 101.31)	83.03 (56.52, 114.80)	< 0.001
Vitamin D (mcg/d)	5.40 (1.90, 13.70)	4.10 (1.60, 11.10)	5.60 (2.20, 14.45)	5.50 (2.05, 14.05)	0.0024
Calcium (mg/d)	918.00 (569.00, 1404.00)	849.00 (569.00, 1250.00)	956.00 (637.00, 1412.00)	991.50 (644.50, 1503.00)	< 0.001
Magnesium (mg/d)	275.00(200.00, 380.00)	264.50 (205.00, 363.00)	304.00 (211.00, 399.00)	318.50 (243.00, 451.00)	< 0.001
Iron (mg/d)	13.31 (9.06, 21.38)	13.69 (9.57, 19.61)	15.18 (10.00, 23.32)	15.40 (10.45, 23.02)	< 0.001
Zinc (mg/d)	11.23 (7.33, 18.96)	10.36 (7.08, 16.10)	12.76 (7.93, 20.26)	12.56 (8.06, 20.68)	< 0.001
Selenium (mg/d)	106.90 (70.70, 160.40)	106.95 (73.30, 147.50)	120.40 (80.30, 171.25)	122.70 (76.90, 170.60)	< 0.001
Urinary iodine (ug/L)	135.60 (72.60, 244.90)	134.75 (70.80, 242.30)	136.00 (82.45, 219.95)	129.70 (68.30, 212.60)	0.207

Data were presented as medians (percentile 25-percentile 75) or frequencies (%)

Participants were clustered into three subgroups according to their demographic and metabolic features, and comparisons of clustering factors are shown in S. Figure 1 and S.Table 1. Group 1 included the eldest and most obese participants, with the highest occurrence of hyperglycemia, hypertension, and CVD. However, Group 1 also had the lowest serum LDL and total cholesterol (S.Table 1), which we speculate might be related to the lipid-lowering drugs commonly used in diabetes and CVD patients. Group 2 had the highest percentage of men, and all participants in this group were current smokers, with 90.24% also having a habit of drinking alcohol. Group 3 was the most metabolically healthy group, with more women, a younger age, and the lowest BMI. None of the participants in Group 3 had hyperglycemia, hypertension, or CVD.

Caffeine intake and thyroid functions also differed significantly among the subgroups, as shown in Table 2. Group 1 had the highest serum TSH levels. In contrast, Group 2 consumed the largest amount of caffeine and, interestingly, had the lowest serum TSH level among the three subgroups. Group 3 had a higher percentage of women and the highest serum levels of autoimmune thyroiditis indicators (TPOAb).

**Table 2:** Thyroid profile and caffeine intake in subgroups

	Subgroups					*p*
	Group 1		Group 2		Group 3	
Thyroid function						
TSH (mU/L)	1.63 (1.14, 2.32)	1.34 (0.94, 1.99)		1.48 (1.04, 2.16)		< 0.001
FT3 (pg/mL)	3.05 (2.82, 3.30)	3.25 (2.99, 3.50)		3.20 (3.00, 3.43)		< 0.001
FT4 (pmol/L)	10.30 (9.30, 11.60)	10.30 (9.00, 11.50)		10.30 (9.20, 11.60)		0.085
TPOAb (U/mL)	0.60 (0.30, 1.50)	0.60 (0.30, 1.20)		0.70 (0.40, 1.50)		0.002
TgAb (U/mL)	0.60 (0.60, 0.60)	0.60 (0.60, 0.60)		0.60 (0.60, 0.60)		0.004
Caffeine intake (mg/d)	87.5 (10.00, 201.00)	147.00 (59.00, 323.00)		85.00 (9.00, 205.00)		< 0.001

Data were presented as medians (percentile 25, percentile 75)

As shown above, we clustered the three subgroups according to the distinct features of the metabolic state, dietary intake, drinking, and smoking habits. Furthermore, thyroid profiles also differed among the groups.

**Fig. 2:**
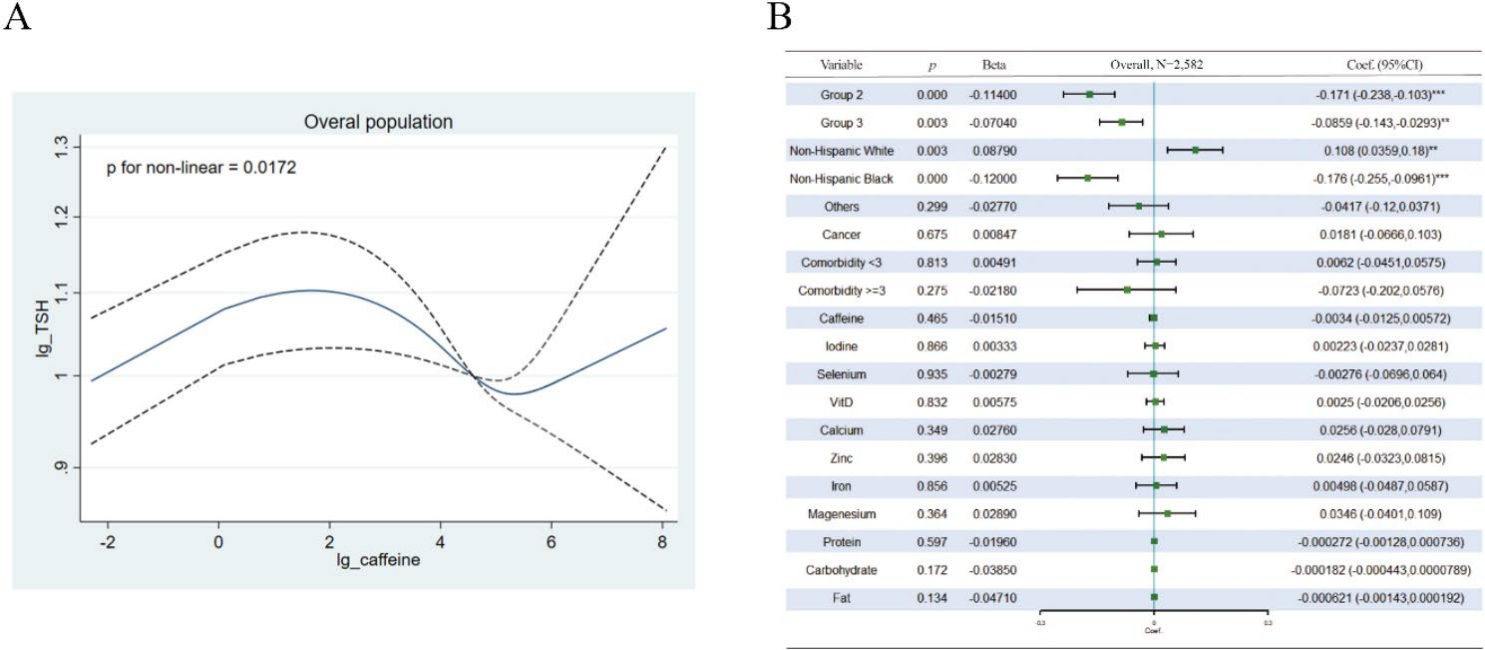
Association between Serum TSH and Caffeine in the Overall Population. (**A**): Nonlinear Relationship Evaluation using the RCS Model. (**B**): Relationship between Serum TSH and Caffeine Intake through Multivariable Linear Regression. Adjustments were made for various factors, including subgroups, race, cancer, comorbidities, urinary iodine, and intake of micronutrients (selenium, vitamin D, calcium, zinc, iron, magnesium) and macronutrients (protein, carbohydrate, and fat). Caffeine, TSH, urinary iodine, and micronutrient intake were log-transformed. A total of 24 subjects were excluded from regression due to missing data in cancer (n = 3) and urinary iodine (n = 21)

To evaluate the association between caffeine intake and TSH levels, we tested their correlation in both the overall group and subgroups. In the overall analysis (Fig. 2), compared to Group 1, being clustered in Groups 2 and 3 was negatively correlated with serum TSH levels (standardized *β* was − 0.114 and − 0.0704, respectively). Non-Hispanic Whites were positively associated with TSH, whereas Non-Hispanic Blacks were negatively associated with TSH compared to Mexican Americans. The overall association between caffeine and TSH levels was not statistically significant. However, the test for nonlinear association was significant (*p* = 0.0172), meaning that the association between them was significantly nonlinear; thus, it would have been incorrect to evaluate their relationship under a linear regression model, as shown in Fig. 2A. Furthermore, as in the overall analysis, TSH levels exhibited strong associations with various metabolic features, and a subgroup analysis was necessary.

**Fig. 3:**
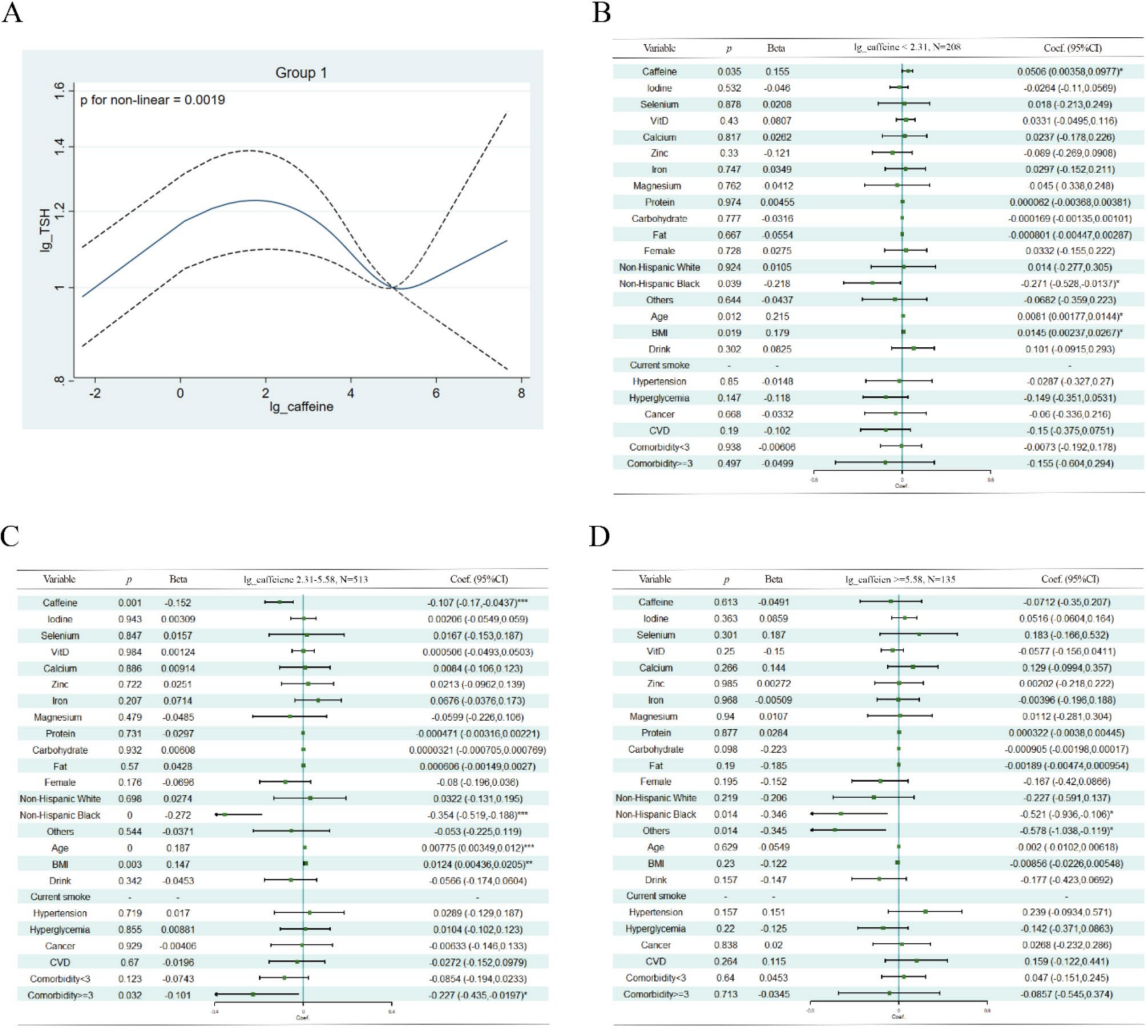
Association between Serum TSH and Caffeine in Group 1. (**A**): Nonlinear Relationship Evaluation using the RCS Model. (**B-D**): Relationship between Serum TSH and Caffeine Intake using Piecewise Linear Regression. Adjustments were made for gender, race, age, BMI, drinking and smoking habits, hyperglycemia, hypertension, cardiovascular disease (CVD), cancer, comorbidities, urinary iodine, and intake of micronutrients (selenium, vitamin D, calcium, zinc, iron, magnesium) and macronutrients (protein, carbohydrate, and fat). Caffeine, TSH, urinary iodine, and micronutrient intake were log-transformed. A total of 12 subjects were excluded from regression due to missing data in cancer (n = 2) and urinary iodine (n = 10)

**Fig. 4:**
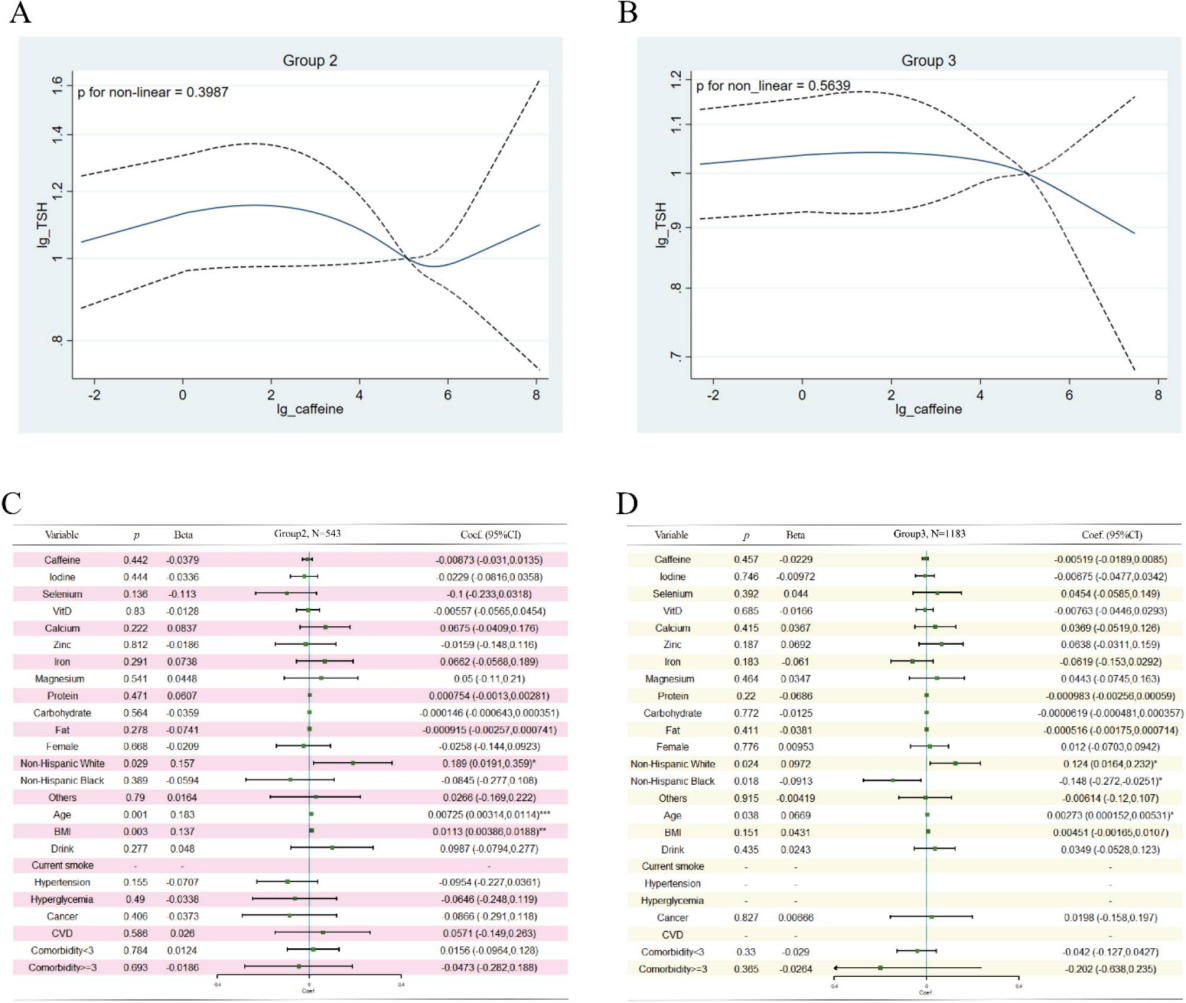
Association between Serum TSH and Caffeine in Group 2 and Group 3. (**A-B**): Nonlinear Relationship Evaluation using the RCS Model. (**C-D**): Relationship between Serum TSH and Caffeine Intake using Multivariable Linear Regression in Group 2 and Group 3. Adjustments were made for race, age, BMI, drinking habits, cancer, comorbidities, urinary iodine, and intake of micronutrients (selenium, vitamin D, calcium, zinc, iron, magnesium) and macronutrients (protein, carbohydrate, and fat). Caffeine, TSH, urinary iodine, and micronutrient intake were log-transformed. A total of 12 subjects were excluded from regression in Group 2 due to missing data on urinary iodine, and 6 subjects were excluded from regression in Group 3 due to missing data in cancer (n = 1) and urinary iodine (n = 5)

In the subgroup analysis, we found that the relationship between caffeine intake and TSH levels was distinctively different among the three groups. Group 1 was identified as the most metabolically unhealthy group, and caffeine intake correlated with TSH nonlinearly (*p* = 0.0019) (Fig. 3A). When caffeine was consumed in minimal amounts (< 9.97 mg/d), its intake was positively associated with TSH levels (*p* = 0.035, standardized *β* = 0.155) after adjusting for age, sex, race, drink, disease state, micronutrients, and macronutrients (Fig. 3B). Interestingly, when consumed in moderate amounts (9.97 ~ 264.97 mg/d), an inverse association was observed (*p* = 0.001, standardized *β* = − 0.152), as shown in Fig. 3C. No association was observed if more than 264.97 mg of caffeine was ingested daily (Fig. 3D). However, in Group 2 and Group 3, no statistically significant association was found between caffeine intake and TSH levels (Fig. 4).

In total, 24 participants were excluded from regression analysis because of missing cancer or urinary iodine values. Multiple imputations were employed using five data sets (S.Table 2), and subsequent linear regression analyses were conducted on the imputed data sets. It is important to note that despite excluding participants with thyroid diseases from the analysis initially, there were still 17 individuals who exhibited abnormal TSH levels (< 0.1 mU/L or > 10 mU/L). Moreover, we performed a sensitivity analysis in which participants with abnormal TSH were further excluded, and the results are shown in S.Table 3. There was no significant association between caffeine and TSH in Group 2 and Group 3. The association between caffeine and TSH was nonlinear in Group 1 (*p* for nonlinear = 0.0061), and when caffeine intake was less than 11.02 mg/d, there was an inverse association between them. The association was positive when caffeine was taken moderately (11.02 ~ 247.15 mg/d). In summary, our sensitivity analysis did not reveal any violations of our previous conclusions.

**Fig. 5:**
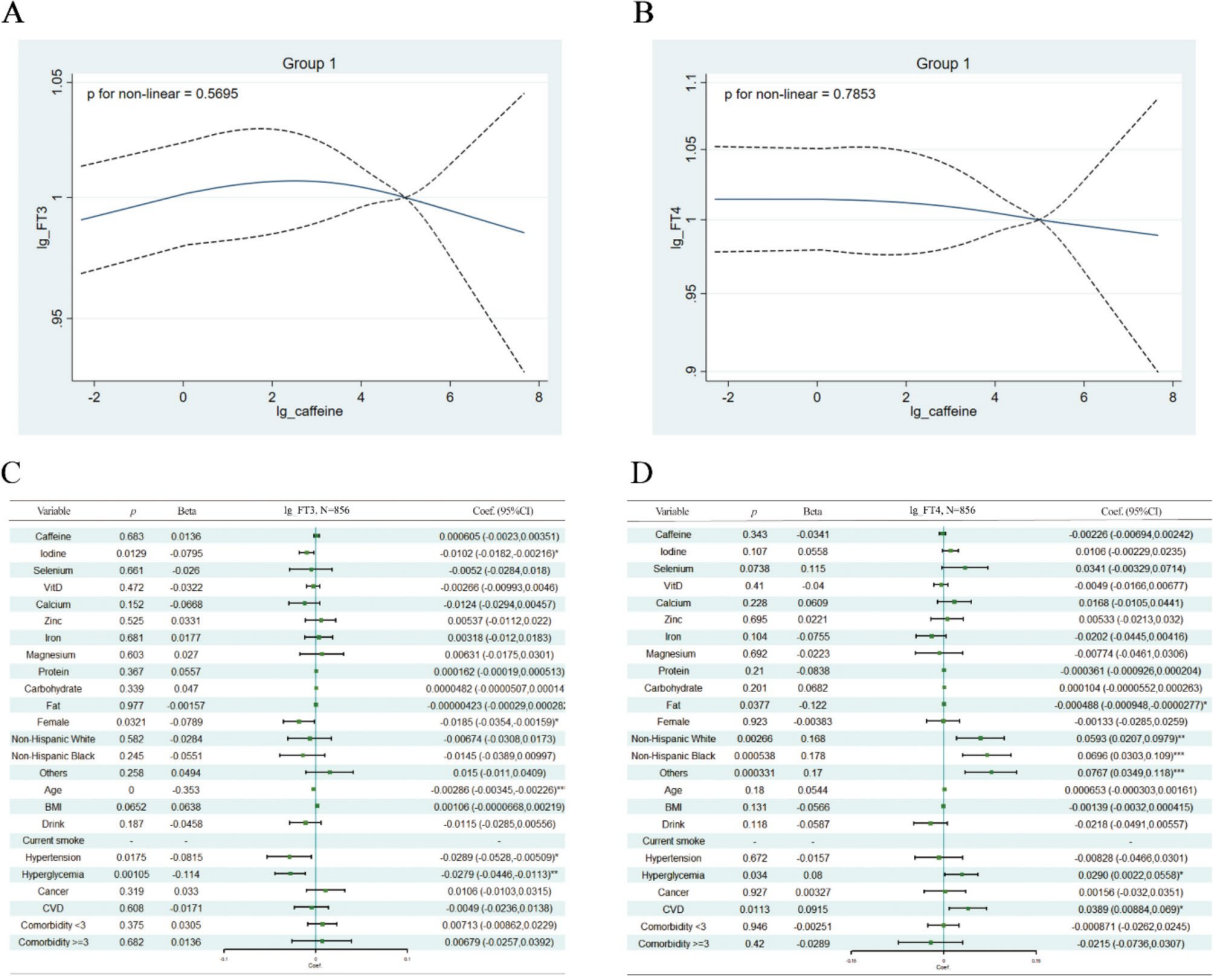
Relationship between Serum FT3 and Serum FT4 with Caffeine Intake in Group 1. (**A-B**): Nonlinear Relationship Evaluation using the RCS Model. (**C-D**): Results of Multivariable Linear Regression Analysis. Adjustments were made for gender, race, age, BMI, drinking and smoking habits, hyperglycemia, hypertension, cardiovascular disease (CVD), cancer, comorbidities, urinary iodine, and intake of micronutrients (selenium, vitamin D, calcium, zinc, iron, magnesium) and macronutrients (protein, carbohydrate, and fat). Caffeine, FT3, FT4, urinary iodine, and micronutrient intake were log-transformed

As TSH is the hormone most sensitive to changes in the thyroid function and the association between caffeine and TSH was found to be significant only in Group 1, we assessed the correlation between caffeine intake and serum FT4 and FT3 levels in Group 1. No statistically significant association was observed between caffeine intake and FT3 or FT4 levels. Serum FT3 and FT4 levels were associated with race and fat ingestion, and FT4 was also associated with CVD (Fig. 5).

## Discussion

In the present study, analysis of the overall population revealed a nonlinear relationship between caffeine intake and serum TSH levels. To control the metabolic cofounders, we classified the participants into three subgroups according to their age, sex, and metabolic characteristics. We found that in the most metabolically unhealthy group, caffeine intake was related to TSH in an inverted V-shaped manner. Caffeine was positively related to TSH levels at minimal doses, while at moderate doses, the association between the two was negative.

The level of caffeine intake differed among the subgroups, and Group 1, identified as the most metabolically unhealthy subgroup, had the lowest caffeine consumption, while Group 2, which had a higher calorie and carbohydrate intake, consumed more caffeine than did the other groups. We speculated that the reason for this diversity might be related to the dietary caffeine sources in Americans. Data from the NHANES 2011–2012 found that among adults, there was a considerable amount of caffeine ingestion from soft drinks and energy drinks (accounting for 17% of the total caffeine intake), which are also high in calories, sugar, and fat, and may lead to excess weight gain [31]. Participants in Group 1 had a higher prevalence of hyperglycemia, CVD, and hypertension, and due to these medical conditions, they may have paid more attention to daily diet control, refraining from soft drinks and excessive sugar intake, which resulted in a lower caffeine intake along with total calorie consumption.

Studies on the direct effects of caffeine on thyroid function are rare. Pietzner demonstrated a strong positive association between serum levels of 3,5-T2 (a metabolite of thyroxine and triiodo-L-thyronine) and serum caffeine metabolites, indicating that thyroid hormones are involved in the possible molecular mechanism underlying the beneficial effects of caffeine [24]. Interventional studies have mainly been performed using animal models. Bartsch reported a transient and non-dose-related increase in FT3 following the administration of medium and high doses of caffeine in Syrian golden hamsters, but the serum TSH levels were not measured [32]. Furthermore, Clozelet al. evaluated the effects of caffeine on newborn rats and found that 10 days of caffeine injections stimulated both T4 and TSH, blunting the pituitary TSH response [33], consistent with our findings. Ahmed et al.also explored the effects of caffeine usage in pregnancy and found that maternal caffeine intake had biphasic effects on thyroid activity, inducing maternal hyperthyroidism and fetal hypothyroidism, which highlights the involvement of the hypothalamic-pituitary-thyroid axis [33]. Caffeine is assumed to modulate pituitary hormone secretion, and this mechanism has been proven to influence the hypothalamic-pituitary-adrenal (HPA) axis [34, 35]. Based on these studies, we speculated that caffeine might impact thyroid activity by affecting the pituitary function. However, the effects of transient and chronic caffeine administration on human thyroid function need to be verified further, and the related mechanisms remain unclear.

Our findings have several clinical implications. Epidemiological studies have reported that approximately 10% of the population is afflicted with subclinical hypothyroidism, with the highest prevalence among women and older adults [17]. Notably, serum TSH levels increase with age in healthy older adults without intrinsic thyroid disease [36], and metabolic disorders can exacerbate this situation by affecting hypothalamic TSH-releasing hormone secretion [37]. Furthermore, higher TSH levels are predictive of persistent subclinical hypothyroidism or progression to overt hypothyroidism in people older than 65 years [38], and the latter has been proven to induce cardiovascular disease and increase all-cause mortality [39]. Inoue et al. recently reported that even moderate TSH elevation (1.94–5.60mU/L) was related to a higher risk of mortality in older adults [40]. Notably, the FDA indicates 400 mg/day of caffeine as safe for healthy adults. In Denmark and the U.K., the recommended upper limit of caffeine intake was also 400 mg/day. In Portugal, this limit is set to 300 mg/day. Our study found a negative association between moderate caffeine intake (9.97–264.97 mg/d) and TSH; this dose fell in the safe range of recommended caffeine intake for healthy adults [9]. Although the causal relationship between caffeine intake and thyroid function requires further verification, as an easily obtainable and widely consumed dietary ingredient, caffeine is a potential candidate for improving thyroid health in people with metabolic disorders.

### Strengths and limitations

This study has two strengths. First, this is the first study on the relationship between caffeine intake and thyroid function, implementing the knowledge of caffeine’s effects on human health. Second, a relatively large sample of participants was analyzed. We clustered the participants into subgroups to minimize the mediating effects of metabolic factors, and RCS analysis was conducted to test possible nonlinear relationships, which made the outcomes more reliable.

However, this study has several limitations. First, as this was a retrospective and observational study, causal relationships could not be explored. Second, a large number of subjects were excluded due to missing data on key variables. Additionally, as medical conditions were self-reported, the recall bias in diet questionnaires was inevitable. However, NHANES applied strict quality control protocols to ensure the quality and authenticity of data. Furthermore, this study put forward the cut-point of 9.97 mg of caffeine intake based on statistical methods, which needed to be validated by more strictly controlled studies.

## Conclusions

Caffeine consumption was correlated with serum TSH nonlinearly, and when taken in moderate amounts (9.97–264.97 mg/d), caffeine demonstrated a positive correlation with serum TSH levels in patients with metabolic disorders.

## Electronic supplementary material

Below is the link to the electronic supplementary material.

Supplementary Material 1

## Data Availability

The original data were retrieved from https://wwwn.cdc.gov/nchs/nhanes/Default.aspx.
