# Supplementary material for: Relationship between caffeine intake and thyroid function: results from NHANES 2007–2012
Source: Nutr J. 2023 Jul 26;22:36. doi: 10.1186/s12937-023-00866-5 (PMC10369722; doi:10.1186/s12937-023-00866-5)
Supplement: Supplementary file 1 — Supplementary Material 1 [file 12937_2023_866_MOESM1_ESM.docx]

**Supplementary materials**

Figure legends

S.Figure 1 Cluster characteristics among subgroups. (A) Distributions of gender, age, BMI, drinking and smoking habits, hyperglycemia, hypertension and CVD. (B-I) Comparisons of gender, age, BMI, drinking and smoking habits, hyperglycemia, hypertension and CVD among subgroups. * *p* < 0.05, compared to group1; *** *p*< 0.001, compared to group1; # *p* < 0.05, compared to group 2; ## *p* < 0.01, compared to group2.


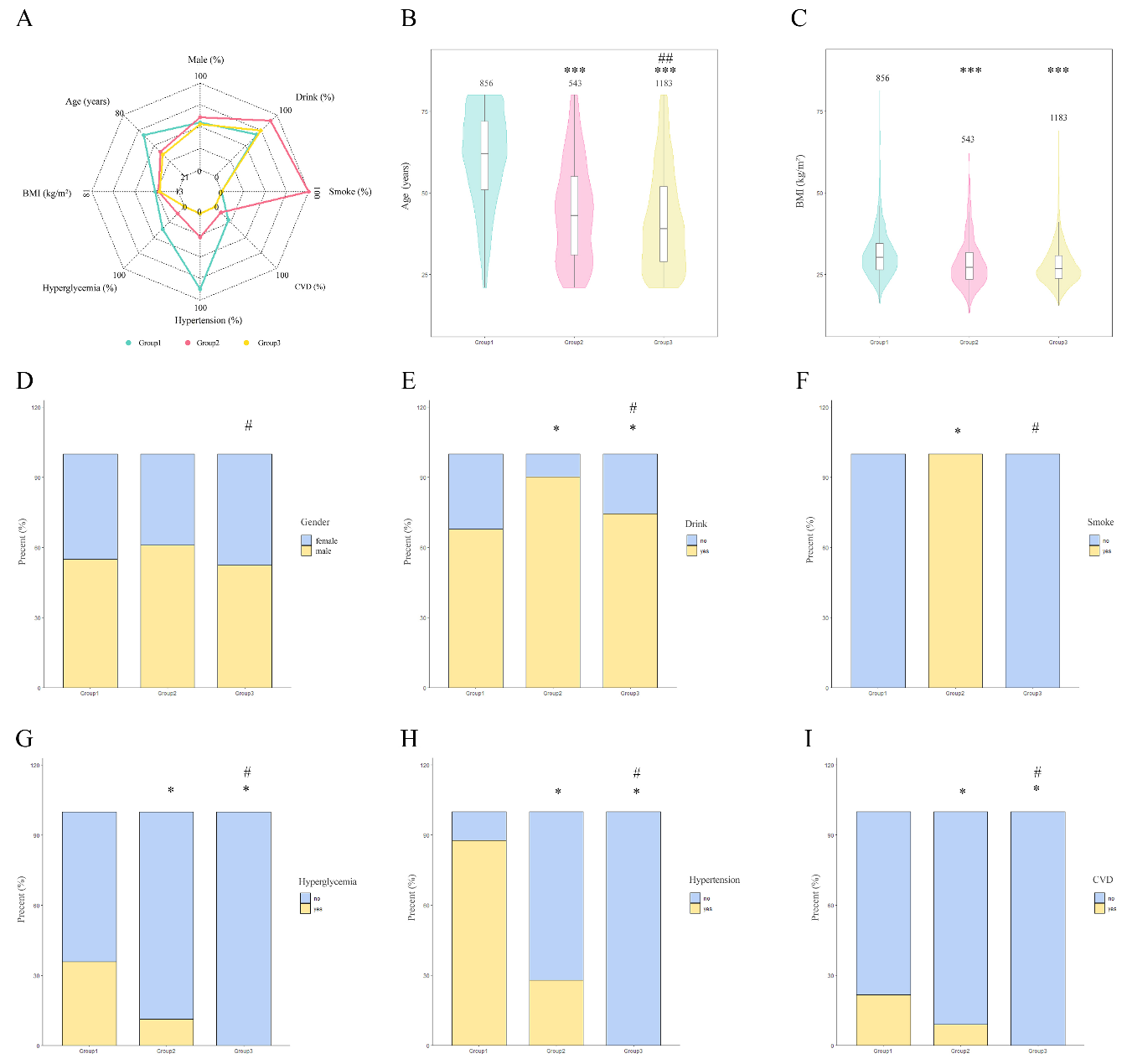


S.Figure 1

S.Table 1 Characteristics of participants clustered in subgroups

|  | Overall population | Subgroups | | | *p* |
| --- | --- | --- | --- | --- | --- |
|  |  | Group 1 | Group 2 | Group 3 |  |
| N | 2582 | 856 | 543 | 1183 |  |
| Age (years) | 48 (34, 62) | 62 (51, 72) | 43 (31, 55) | 39 (29, 52) | <0.001 |
| Sex |  |  |  |  |  |
| male (%) | 1421 (55.0) | 470 (18.2) | 331 (12.8) | 620 (24.0) | 0.004 |
| female (%) | 1161 (45.0) | 386 (14.9) | 212 (8.2) | 563 (21.8) |  |
| Race/Ethnicity |  |  |  |  |  |
| Mexican American (%) | 383 (14.83) | 117 (13.67) | 62 (11.42) | 204 (17.24) | <0.001 |
| Non-Hispanic White (%) | 1097 (42.49) | 352 (41.12) | 264 (48.62) | 481 (40.66) |  |
| Non-Hispanic Black (%) | 574 (22.23) | 232 (27.1) | 127 (23.39) | 215 (18.17) |  |
| Other races | 528 (20.45) | 155 (18.11) | 90 (16.57) | 283 (23.92) |  |
| Education |  |  |  |  |  |
| > High school (%) | 1338 (51.9) | 411 (15.9) | 201 (7.8) | 726 (28.2) | <0.001 |
| <= High school (%) | 1241 (48.1) | 443 (17.2) | 342 (13.3) | 456 (17.7) |  |
| Marital Status |  |  |  |  |  |
| Married/Living with partner (%) | 1548 (60.0) | 517 (20.0) | 295 (11.4) | 736 (28.5) | <0.001 |
| Widowed/Separated/Divorced (%) | 547 (21.2) | 248 (9.6) | 140 (5.4) | 159 (6.2) |  |
| Never married (%) | 487 (18.9) | 91 (3.5) | 108 (4.2) | 288 (11.2) |  |
| Smoke |  |  |  |  |  |
| < 100 cigarretes (%) | 543 (21.0) | 0 (0.0) | 543 (21.0) | 0 (0.0) | <0.001 |
| >= 100 cigarretes (%) | 1393 (54.0) | 498 (19.3) | 0 (0.0) | 895 (34.7) |  |
| Drink |  |  |  |  |  |
| < 12 drinks/year (%) | 1951 (75.6) | 580 (22.5) | 490 (19.0) | 881 (34.1) | <0.001 |
| >= 12 drinks/year (%) | 631 (24.4) | 276 (10.7) | 53 (2.1) | 302 (11.7) |  |
| BMI (kg/m2) | 27.86 (24.3, 32.4) | 30.20 (26.45, 34.55) | 27.22 (23.49, 31.80) | 26.70 (23.78, 30.89) | <0.001 |
| Hyperglycemia (%) | 367 (14.21) | 306 (35.75) | 61 (11.23) | 0 (0) | <0.001 |
| Hypertension (%) | 902 (34.93) | 751 (87.73) | 151 (27.81) | 0 (0) |  |
| CVD (yes, %) | 232 (8.99) | 184 (21.50) | 48 (8.84) | 0 (0) |  |
| Cancer (yes, %) | 228 (8.84) | 133 (15.57) | 40 (7.37) | 55 (4.65) | <0.001 |
| Comorbidities |  |  |  |  |  |
| 0 (%) | 1542 (59.72) | 348 (40.65) | 316 (58.2) | 878 (74.22) | <0.001 |
| <3 (%) | 950 (36.79) | 459 (53.62) | 194 (35.73) | 297 (25.11) |  |
| >=3 (%) | 90 (3.49) | 49 (5.72) | 33 (6.08) | 8 (0.68) |  |
| Serum lipids |  |  |  |  |  |
| LDL (mmol/L) | 2.97 (2.38, 3.62) | 2.83 (2.22, 3.48) | 3.00 (2.38, 3.67) | 3.05 (2.46, 3.65) | 0.002 |
| HDL (mmol/L) | 1.27 (1.06, 1.55) | 1.26 (1.06, 1.52) | 1.19 (1.01, 1.47) | 1.34 (1.11, 1.63) | <0.001 |
| Total cholesterol (mmol/L) | 4.98 (4.29, 5.72) | 4.89 (4.16, 5.64) | 4.99 (4.29, 5.84) | 5.02 (4.34, 5.72) | 0.029 |
| Energy (kcal/d) | 1977.50  (1466.00, 2633.00) | 1813.50  (1319.00, 2328.00) | 2161.00  (1580.00, 2848.00) | 2048  (1532.00, 2702.00) | <0.001 |
| Protein (gm/d) | 75.86 (52.62, 102.96) | 70.84 (50.53, 95.60) | 75.05 (53.18, 106.67) | 78.60 (54.73, 106.62) | 0.001 |
| Carbohydrate (gm/d) | 240.88 (176.45, 323.40) | 233.00 (158.89, 288.24) | 253.75 (181.20, 348.97) | 252.47 (188.89, 339.20) | <0.001 |
| Fat (gm/d) | 71.16 (47.77, 103.42) | 66.19 (42.68, 94.89) | 74.10 (51.53, 106.95) | 73.63 (49.36, 106.17) | <0.001 |
| Vitamin D (mcg/d) | 5.10 (2.00, 13.40) | 6.30 (2.30, 16.60) | 3.50 (1.20, 9.70) | 5.00 (2.10, 12.40) | <0.001 |
| Calcium (mg/d) | 926.00  (606.00, 1410.00) | 916.00  (600.00, 1411.50) | 838.00  (509.00, 1324.00) | 965.00  (646.00, 1456.00) | <0.001 |
| Magnesium (mg/d) | 290.00 (211.00, 399.00) | 283.00 (207.00, 390.50) | 269.00 (190.00, 392.00) | 300.00 (226.00, 409.00) | <0.001 |
| Iron (mg/d) | 14.32 (9.73, 21.91) | 13.97 (9.66, 21.53) | 13.48 (8.96, 21.61) | 14.72 (10.19, 22.80) | 0.004 |
| Zinc (mg/d) | 11.62 (7.51, 19.07) | 12.22 (7.58, 20.14) | 10.72 (7.00, 17.35) | 11.62 (7.65, 18.82) | 0.005 |
| Selenium (mg/d) | 114.70 (75.10, 161.50) | 114.45 (76.55, 162.55) | 109.10 (70.90, 162.40) | 116.30 (75.80, 160.80) | 0.157 |
| Urinary iodine (ug/L) | 134.00 (74.20, 230.60) | 150.15 (87.10, 248.60) | 130.60 (72.00, 217.70) | 124.30 (65.20, 222.30) | <0.001 |

Data were presented as medians (percentile 25-percentile 75) or frequency (%).

S.Table 2 Association of caffeine and serum TSH levels before and after Multiple imputation (MI)

| Population | Before MI | M1 | M2 | M3 | M4 | M5 |
| --- | --- | --- | --- | --- | --- | --- |
| Overall | -0.0151 (0.465) | -0.0130  (0.526) | -0.0131  (0.524) | -0.0129  (0.529) | -0.0132  (0.522) | -0.0131  (0.523) |
| Group1 |  |  |  |  |  |  |
| <2.31 | 0.196  (0.008) | 0.189  (0.009) | 0.189  (0.009) | 0.189  (0.009) | 0.189  (0.009) | 0.189  (0.009) |
| 2.31-5.58 | -0.152  (0.001) | -0.154  (0.001) | -0.154  (0.001) | -0.158  (0.001) | -0.153  (0.001) | -0.154  (0.001) |
| >=5.58 | -0.0491  (0.613) | 0.0109  (0.908) | 0.123  (0.897) | 0.144  (0.879) | 0.00842  (0.929) | 0.0132  (0.888) |
| Group2 | -0.0222  (0.650) | -0.0191  (0.694) | -0.0193  (0.692) | -0.0182  (0.709) | -0.0198  (0.683) | -0.0193  (0.691) |
| Group3 | -0.0157  (0.609) | -0.00979  (0.748) | -0.00979  (0.749) | -0.00979  (0.749) | -0.00979  (0.519) | -0.00980  (0.748) |

MI: multiple imputation; the association of caffeine intake and serum TSH levels are represented as standardized beta (*p* value); caffeine intake and TSH were log-transformed. M1-M5 refers to the 5 subsets of multiple imputation. Regressions were adjusted for sex, age, race, medical conditions, micronutrient and macronutrient intake.

S.table 3 Relationship between caffeine intake and TSH

| Population | N | *P* for non-linear | Beta (*P*) |
| --- | --- | --- | --- |
| Overall | 2,565 | 0.0231 | -0.00681 (0.742) |
| Group1 | 851 | 0.0061 | 0.0115 (0.784) |
| <2.41 | 221 | / | 0.146 (0.044) |
| 2.41-5.52 | 485 | / | -0.130 (0.006) |
| >=5.52 | 145 | / | -0.0597 (0.590) |
| Group2 | 540 | 0.2956 | -0.0244 (0.620) |
| Group3 | 1,174 | 0.7087 | -0.00353 (0.909) |

Caffeine intake and TSH were log-transformed. Regressions were adjusted for sex, age, race, medical conditions, micronutrient and macronutrient intake.
